# Screening for hemoglobin disorders and investigating their hematological and demographic profile among patients attending a tertiary-care hospital in southern India—a descriptive study

**DOI:** 10.1097/j.pbj.0000000000000271

**Published:** 2024-10-23

**Authors:** Dheebika Kuppusamy, Kolar Vishwanath Vinod, Rakhee Kar

**Affiliations:** a Departments of Pathology,; b Medicine, Jawaharlal Institute of Postgraduate Medical Education and Research (JIPMER), Puducherry, India

**Keywords:** hemoglobinopathy, Hb HPLC, screening, southern India, thalassemia

## Abstract

**Background:**

Hemoglobinopathies and thalassemias are widely prevalent autosomal inherited recessive disorders of the structure and synthesis of hemoglobin, respectively. Given the regional heterogeneity of these disorders, this study was undertaken to elucidate the patterns and prevalence of these disorders from this region.

**Methods:**

This was a tertiary-care hospital-based study in southern India over 4 years. Screening for hemoglobin (Hb) disorders was done using Hb high-performance liquid chromatography in patients based on initial screening of complete blood count parameters and for clinically indicated cases.

**Results:**

A normal Hb HPLC pattern was observed in 404 (72.1%) and abnormal in 156 (27.9%) of 560 cases studied. The abnormalities seen were heterozygous β-thalassemia in 73 (46.8%), homozygous β-thalassemia in 19 (12.2%), heterozygous α-thalassemia in 7 (4.5%), HbH disease and heterozygous δβ-thalassemia in 1 (0.6%) each, sickle cell trait in 9 (5.8%), sickle cell anemia in 8 (5.1%), sickle β-thalassemia in 17 (10.9%), HbS+ Hb D-Punjab in 1 (0.6%), heterozygous HbE in 6 (3.8%), homozygous HbE in 2 (1.3%), HbE β-thalassemia in 3 (1.9%), Hb J-Meerut in 1 (0.6%), Hb Kirksey in 4 (2.6%), unknown α-hemoglobinopathy in 2 (1.3%), and Hb Lepore in 2 (1.3%) cases. Most of the patients were from the neighboring districts, and some were referred from other states.

**Conclusion:**

The most common hemoglobin disorders were heterozygous β-thalassemia in 73 cases (46.8%) and sickle hemoglobinopathy in 35 cases (22.4%). A heterogeneous group of hemoglobin disorders, including uncommon α-hemoglobinopathies, was found in the study population, likely due to the referral of patients from various regions.

## Introduction

Hemoglobinopathies and thalassemias are the most common monogenic and autosomal inherited recessive disorders of the structure and synthesis of hemoglobin. In addition to being prevalent in the Mediterranean region, Southeast Asia, the Indian subcontinent, and the Middle East, β-thalassemias are now a global problem that has spread to most of Europe, the Americas, Australia, and many other countries because of migration.^1,2^ Around 7% of the global population carries an abnormal hemoglobin gene. Approximately 80% of newborns with these diseases are born each year in low-income or middle-income nations. Approximately 70% are born with sickle cell disease (SCD) and the rest with thalassemia syndromes.^3^

India has surpassed China as the most populous country in the world with approximately 1.4 billion population. India's population is exceptionally diverse because of its geography, ethnicity, religion, and language variations.^4^ Approximately 8.6% of the population belongs to indigenous communities; many are socioeconomically underprivileged and live in remote areas.^5^ Estimates suggest that nearly 3–4% of the population in India are thalassemia carriers, which would be 30-40 million carriers.^3^ Several epidemiological studies have shown many nontribal and tribal communities where the prevalence of β-thalassemia trait is higher (5.3–17.0%) than the average of 3–4% projected for the entire country. Hemoglobin E (HbE) is prevalent in northeastern and eastern India and sickle hemoglobin (HbS) in central India and parts of western, eastern, and southern India.^6^

The health burden of hemoglobinopathies is huge because of the size and genetic complexity of the population. This makes it necessary to study the prevalence of hemoglobin disorders in a diversified populated country like India. Regional heterogeneity highlights the importance of understanding the local disease burden and setting goals and targets while considering such patterns. In this regard, screening with hemoglobin high-performance liquid chromatography (Hb HPLC) is an effective tool for detecting hemoglobin variants.^7^ This study aimed to screen for thalassemia and hemoglobinopathies in a referral hospital-based population in southern India and map the prevalence with the regions from which these patients were referred.

## Materials and methods

The study was a descriptive cross-sectional analysis conducted at a tertiary-care referral center in southern India spanning 4 years from January 2019 to December 2022. This study was conducted in the Hematology section of the Department of Pathology and approved by the institute’s ethics committee (JIP/IEC/2018/0283). Screening for thalassemia and hemoglobinopathy was done using Hb HPLC in patients either based on initial screening of complete blood count (CBC) parameters with red cell indices suggestive of thalassemia or hemoglobinopathy or for clinically suspected cases referred as such to the laboratory.

Samples collected in EDTA tubes were processed for CBC in an automated blood cell counter (Sysmex Corporation, XT4000i, Kobe, Japan). In indicated cases, Hb HPLC (D10, Biorad, California) analysis was done, and based on the fractions of hemoglobins, their retention time (RT), and peak characteristics, normal and variant hemoglobins were identified. Family screening was also done for some cases. All the positive cases were further subgrouped based on the type of thalassemia and hemoglobinopathy.

The hematological parameters from CBC, like hemoglobin (Hb), and red blood cell (RBC) indices, hematocrit (HCT), mean corpuscular volume (MCV), mean corpuscular hemoglobin (MCH), mean corpuscular hemoglobin concentration (MCHC), and red blood cell distribution width (RDW), were noted. The fractions of various Hb variants, such as A_0_, A_2_, F, and S, and other variants, along with their RT, were recorded from the chromatogram.

Wherever possible, a detailed medical history was taken, and in other cases, the available clinical data from the request form were collated. This included age, gender, ethnic origin, consanguineous marriage, history of blood transfusion, age at first presentation, size of the liver and spleen, and frequency of blood transfusions. All these are presented using summary statistics. The region to which these patients belonged was retrieved from the medical records.

## Results

In 4 years, encompassing the COVID-19 pandemic, 560 cases underwent screening for hemoglobinopathies and thalassemia. The patients presented were in a wide range of age groups from 6 months to 64 years. Positive screening in the form of hemoglobin defect, either thalassemia or hemoglobinopathy, was identified in 156 cases (27.9%). The breakdown of the cases is presented in Table 1.

**Table 1:** Breakdown of cases based on diagnosis of hemoglobinopathies detected by hemoglobin HPLC and percentage of abnormality among total cases screened.

Diagnosis	No. of positive cases (n = 156)	Percentage among positive cases (n = 156)	Percentage among screened cases (n = 560)
Heterozygous β-thalassemia	73	46.8	13
Homozygous β-thalassemia	19	12.2	3.4
Heterozygous α-thalassemia	7	4.5	1.3
HbH disease	1	0.6	0.2
Heterozygous δβ-thalassemia	1	0.6	0.2
Sickle cell trait (Heterozygous HbS)	9	5.8	1.6
Sickle cell anemia (homozygous HbS)	8	5.1	1.4
Sickle β-thalassemia	17	10.9	3
HbS + Hb D-Punjab	1	0.6	0.2
HbE trait (heterozygous HbE)	6	3.8	1.1
HbE disease (homozygous HbE)	2	1.3	0.4
HbE β-thalassemia	3	1.9	0.5
Hb J-Meerut	1	0.6	0.2
Hb Kirksey	4	2.6	0.7
Unknown α-hemoglobinopathy	2	1.3	0.4
Hb Lepore	2	1.3	0.4

Hb, hemoglobin; HbS, hemoglobin sickle; HbE,hemoglobin E; HbH, hemoglobin H.

Of the 156 cases diagnosed, heterozygous β-thalassemia was the most common abnormality detected, accounting for 73 cases (46.8%), while the commonest hemoglobinopathy observed was sickle hemoglobinopathy. A total of 19 cases of homozygous β-thalassemia accounting for 12.2% were identified. Most cases of thalassemia major present within the first 2 years of life. The predominant features of the blood profile were severe anemia, anisopoikilocytosis, and a microcytic hypochromic appearance accompanied by numerous nucleated RBCs.

Sickle hemoglobinopathy was diagnosed in 35 cases (22.4%). Homozygous HbS was diagnosed in 8 cases (5.1%) presented with an average HbS fraction (57.46%). The blood picture showed target cells, occasional nRBC, and a few irreversible sickle cells in the peripheral smear. 9 cases were diagnosed with sickle cell trait (5.8%) with an average HbS fraction in the sickle window of 32.9%. Sickle β-thalassemia was confirmed in 17 cases with an average HbS window of 56.92%. One case of HbS +Hb D-Punjab was seen, which had an unknown peak of 42.9% at RT of 3.88 minutes corresponding to Hb D-Punjab, along with a HbS window (34.9%); HbF was elevated (14.3%), and HbA_2_ was reduced (1.6%).

The HbE hemoglobinopathies comprised 6 heterozygous cases (3.8%), 2 homozygous cases (1.3%), and 3 HbE β-thalassemia compound heterozygous cases (1.9%). The average HbE fraction was 24.36%, 79.9%, and 40.1%, respectively, with the latter demonstrating an elevated proportion of HbF of 29.4%.

We observed 7 cases of heterozygous α-thalassemia (4.5%) with an average HbA_2_ level of 1.69%. We also had single cases each of δβ-thalassemia trait and HbH disease. δβ-thalassemia case presented with increased HbF (14.9%) and decreased HbA_2_ (1.9%). HbH diagnosed had an unknown preintegration peak of 11.6%.

One case with Hb J-Meerut with coexisting nutritional deficiency presented with severe anemia with a Hb HPLC pattern showing decreased HbA_2_ of 1.8% and an unknown window (P3) peak of 17% with an RT of 1.47 min. Two cases of hemoglobin Lepore were identified, accounting for 1.3%, showing an elevated HbA_2_ region peak of 12.8%, and presented with mild anemia.

A rare case of alpha hemoglobinopathy in 4 family members with an unknown peak of 16.8% at retention time (RT) of 4.34 minutes presenting with low SpO2 conforming with Hb Kirksey was identified, constituting 2.6% of the total cases. Another family of 2 members (father and son) (1.3%) showed an unknown peak (14.9%) at an RT of 3.9 minutes. Although the RT corresponds to the D-window, the proportion of variant hemoglobin being lower than expected for β-hemoglobinopathy made us consider this an unknown α-hemoglobin variant.

**Figure 1.**
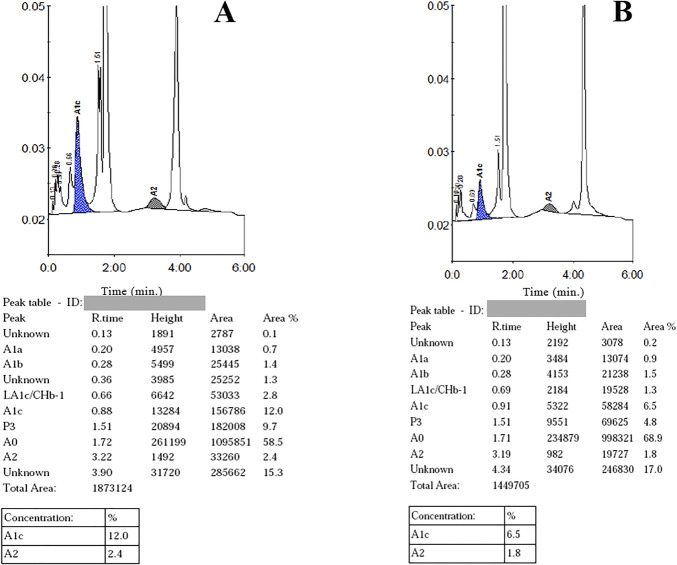
Chromatogram of abnormal hemoglobin variants: unknown α-hemoglobinopathy (A) showing an unknown peak at a retention time of 3.90 minutes with a peak area of 15.3% and hemoglobin Kirksey (B) with an unknown peak at a retention time of 4.34 minutes and peak area of 17.0%. “Time (min.)” indicates the retention time in minutes for each fraction to elute while “%” denotes the proportion of hemoglobin present in each elution peak

The hematological findings, including hemoglobin HPLC values of each of the groups mentioned above, are summarized in Table 2. A few representative chromatograms of uncommon cases (α-hemoglobinopathies) are given in Figure 1.

**Table 2:** Summary of mean hematological and hemoglobin HPLC findings of thalassemia and hemoglobinopathies.

SI no.	Diagnosis	No. of cases	Hb (g/dl)	RBC count (mil/cumm)	HCT (%)	MCV (fl)	MCH (ng)	MCHC (gm %)	RDW (%)	HbF (%)	HbA_0_ (%)	HbA_2_ (%)	HbS (%)	Unknown peak (%)/RT (min)
1	Heterozygous β-thalassemia	73	10.6	5.1	39.68	65.63	20.5	31.8	18.89	1.4	78.97	5.29		
2	Homozygous β-thalassemia	19	7.03	3.09		70.69	23.68	32.75	28.64	46.45	35.97	5.52		
3	Heterozygous α-thalassemia	7	10.99	5.7	36.5	64.5	19.29	29.99		1.62	82.04	1.69		
4	HbH disease	1	8.5	4.5	31.1	68.8	18.8	27.3	30.4	10.7	70.2	1.8		11.6/preintegration peak
5	Heterozygous δβ-thalassemia	1	10.1	5.4	31.1	57.2	18.6	32.5	23.5	14.9	72.1	1.9		
6	Sickle cell heterozygous	9	11.43	4.26	33.68	79.55	26.53	33.93	17.18	0.92	55.5	3.18	32.91	
7	Sickle cell anemia (homozygous)	8	7.74	2.84	23.4	83.69	27.73	33.03	21.68	16.64	7.31	2.24	57.46	
8	Sickle β-thalassemia	17	8.17	3.74	26.53	66.93	22.79	30.69	23.87	16.66	16.29	4.62	56.92	
9	HbS+ Hb D-Punjab	1	10.3	3.1	29.9	96.5	33.2	34.4		14.3	2.3	1.6	34.9	42.9/3.8
10	Heterozygous HbE	6	9.3	4.3	25.77	68.84	21.18	30.6	19.76	1.08	60.78	24.36		
11	Homozygous HbE	2	12.1	5.9	37.5	63.7	20.5	32.3	16	1.2	5.8	79.9		
12	HbE β-thalassemia	3	6.9	3.32	22.5	68.8	21.1	30.6	29.1	29.4	17.6	40.1		
13	Hb J-Meerut	1	2.7	1.96	11.2	57.1	13.8	24.1	34.5	1.9	71	1.8		17/1.47
14	Hb Kirksey	4	11.1	3.8	34.2	88.6	28.9	32.6	14.2	1.3	69.2	2.2		16.8/4.34
15	Unknown α-hemoglobinopathy	2	11.1	4.1	29.5	77.6	27	34.3	16.4	1.8	64	3.4		14.9/3.9
16	Hb Lepore	2	7.6	4.75	23.3	49.1	16	32.6		11.7	65.1	12.8		

Hb, hemoglobin; RBC, red blood cell; HCT, hematocrit; MCV, mean corpuscular volume; MCH, mean corpuscular Hb; MCHC, mean corpuscular Hb concentration; RDW, red blood cell distribution width; RT, retention time.

Although most of the cases were from the Villupuram and Cuddalore districts of Tamil Nadu, partly because of the hospital's proximity, we also had cases from the Puducherry union territory (UT). In addition, some of the cases were referred from other states, notably West Bengal and Odisha. The case breakdown and regional distribution are given in Table 3.

**Table 3:** Distribution of diagnosed cases of thalassemias and hemoglobinopathies based on their region.

Diagnosis	No. of cases	Puducherry	Villupuram	Cuddalore	Nammakal	Thiruvannamalai	Dindigul	Erode	Trichy	Dharmapuri	Karur	Thiruvarur	Krishnagiri	Mayiladuthurai	Salem	Kallakurichi	Vellore	Others
Heterozygous β-thalassemia	73	16 (21.9)	12 (16.4)	8 (10.9)	5 (6.8)	3 (4.1)	3 (4.1)	1 (1.3)	1 (1.3)	1 (1.37)	1 (1.3)	1 (1.3)	0	0	2 (2.7)	2 (2.7)	2 (2.7)	15 (20.6)
Homozygous β-thalassemia	19	1 (5.36)	4 (5.5)	2 (10.5)	1 (5.3)	6 (31.6)	1 (5.3)			1 (5.26)			0	0	2 (10.5)		1 (5.3)	
Heterozygous α-thalassemia	7	2 (28.6)	2 (28.6)										1 (14.3)					2 (28.6)
HbH disease	1	1 (100)																
Heterozygous δβ-thalassemia	1	1 (100)																
Heterozygous HbS	9		3 (3.3)			1 (11.1)				1 (11.1)								4 (44.4)
Homozygous HbS	8		1 (12.5)	1 (12.5)	1 (12.5)	1 (12.5)				1 (12.5)					1 (12.5)	1 (12.5)		1 (12.5)
Sickle β-thalassemia	17		6 (35.2)		4 (23.5)								1 (5.9)			1 (5.9)		5 (29.4)
HbS + Hb D-Punjab	1		0															1 (100)
Heterozygous HbE	6	1 (16.7)												1 (16.7)				4 (66.8)
Homozygous HbE	2																	2 (100)
HbE β-thalassemia	3		0	1 (33.3)														2 (66.7)
Hb J-Meerut	1		1 (100)															
Hb Kirksey	4			4 (100)														
Unknown α-hemoglobinopathy	2	2 (100)																
Hb Lepore	2		1 (50)												1 (50)			
Total	156																	

The percentage of thalassemias and hemoglobinopathies of each region is given in parentheses.

## Discussion

Screening for hemoglobinopathies and thalassemia is vital in a highly populated country like India. A complete hemogram combined with Hb HPLC is a reliable, robust, and rapid technique for screening thalassemias and hemoglobinopathies.^7,8^ India has reported a few extensive studies on Hb HPLC analysis to identify hemoglobinopathies in hospitals and community-based screening programs from various parts of the country^9,10^ and southern India.^11-13^ However, there is a paucity of data from this region, especially the Puducherry UT.

In this study, the most common hemoglobin disorders observed were β-thalassemia trait in 73 cases (46.8%) and sickle hemoglobinopathy in 35 cases (22.4%). Besides β-thalassemia syndromes and HbS disorders, we identified several other types of hemoglobin abnormalities in our study that included a few cases of HbE hemoglobinopathies, HbH disease, and heterozygous δβ-thalassemia. 3 unusual alpha-chain variants were identified in 7 individuals, including Hb J-Meerut, Hb Kirksey, and unknown alpha-hemoglobinopathy. A double heterozygote case of HbS + Hb D-Punjab was also reported.

The prevalence of β-thalassemia trait and other hemoglobinopathies varies in different parts of the country. In a multicenter study conducted on pregnant women and students, spanning 6 cities of India, namely Maharashtra, Gujarat, West Bengal, Assam, Karnataka, and Punjab, an overall prevalence of 2.78% for β-thalassemia trait was reported, with variations ranging from 1.48% to 3.64% across different states. The study encompassed 100 distinct ethnic groups representing diverse populations.^9^ In a study conducted in Kerala, thalassemia major and intermedia constituted approximately 3% of the tribal population and HbD and HbE constituted 3% and 2% of the cases.^11^ The Institute of Genetics & Hospital for Genetic Diseases, Andhra Pradesh, conducted screening in a total of 1592 suspected cases of anemia; 7.47% were found to be thalassemia major, 21.7% were thalassemia carriers, 3.3% sickle cell anemia, 2.4% sickle cell trait, 1.69% HbE, 0.56% HbD, and 1.5% sickle β-thalassemia.^12^ In a 1-year study conducted by Apollo Hospitals in Chennai, a total of 996 cases screened for thalassemia and other hemoglobinopathies and 543 abnormal chromatograms were seen, among which the most typical disorder they encountered was β-thalassemia trait (37.9%), followed by HbE trait (23.2%), homozygous HbE disease (18.9%), HbS trait (5.3%), HbE β-thalassemia (4.6%), HbS β-thalassemia (2.5%), β-thalassemia major (2.3%), HbH (1.6%), homozygous HbS (1.4%), and HbD trait (0.7%).^13^ This reported prevalence is similar to our study in terms of β-thalassemia trait, but they had more frequency of HbE disorders while we encountered more HbS disorders.

However, these hospital-based screening studies like ours cannot be taken as indicators of population prevalence. The widely varying prevalence estimates of hemoglobin disorders are due to the wide disparity in the denominator in various studies. There are two major reasons for that. The first is inherent in the inclusion criteria wherein some studies have included all samples sent for Hb HPLC. By contrast, others have purposive sampling such as clinically indicated cases and suggestive screening CBC/hemogram parameters like our study. The other factor is the reporting of prevalence among hemoglobin disorders encountered versus among all cases screened. We have indicated both, as given in Table 1.

Our study also revealed some rare α-hemoglobinopathies. A case of Hb Kirksey was identified in an elderly woman referred for evaluation of low oxygen saturation (SpO2) levels, also seen in her sisters and niece. She was otherwise asymptomatic. Hb Kirksey is a known low oxygen affinity Hb variant caused by point mutation of the alpha-2 gene (HBA2), at codon 94 (GAC to GTC), resulting in a substitution of aspartic acid to valine.^14^ The discrepancy between SpO2 and partial pressure of arterial oxygen (PaO2) detected on blood gas analysis often provides a clue to the diagnosis as was also seen in our case.^15^ The other case we encountered was seen in a 27-year-old and his father, otherwise asymptomatic, with an abnormal peak of 15.3% at RT of 3.9 minutes. While the retention time aligned with that of Hb D-Punjab, the unusually lower abnormal fraction compared with what would typically be expected in a β-hemoglobin variant prompted us to consider the possibility of an unknown α-hemoglobinopathy in this patient. However, we were unable to characterize these further.

Future population screening procedures for hemoglobinopathy may change if newer technologies are validated and incorporated into hemoglobinopathy testing standards. Given the high prevalence of β-thalassemia trait in the Indian subcontinent, this is particularly crucial. In conclusion, heterozygous β-thalassemia and HbS disorders were the major abnormalities diagnosed in our study. We also observed specific uncommon α-hemoglobin variants. Numerous cases originated from neighboring districts and various other states, likely attributed to proximity and referrals, respectively.
